# Effect of pH Cycling Frequency on Glass–Ceramic Corrosion

**DOI:** 10.3390/ma13163655

**Published:** 2020-08-18

**Authors:** Shu-Min Hsu, Fan Ren, Christopher D. Batich, Arthur E. Clark, Dan Neal, Josephine F. Esquivel-Upshaw

**Affiliations:** 1 Department of Restorative Dental Sciences, Division of Prosthodontics, University of Florida College of Dentistry, Gainesville, FL 32610, USA; shuminhsu@ufl.edu (S.-M.H.); BCLARK@dental.ufl.edu (A.E.C.); 2 Department of Chemical Engineering, University of Florida Herbert Wertheim College of Engineering, Gainesville, FL 32611, USA; fren@che.ufl.edu; 3 Department of Materials Science and Engineering, University of Florida Herbert Wertheim College of Engineering, Gainesville, FL 32611, USA; cbati@mse.ufl.edu; 4 Department of Surgery, University of Florida College of Medicine, Gainesville, FL 32610, USA; dneal@ufl.edu

**Keywords:** glass–ceramic, pH, corrosion, weight loss, ion release

## Abstract

The effect of pH changes on the chemical durability of dental glass–ceramic materials was evaluated using weight loss and ion release levels. The hypothesis that increased pH changes will exhibit greater corrosion was investigated. The ion concentration was analyzed using inductively coupled plasma atomic emission spectrometer (ICP). The surface compositions were investigated using X-ray photoelectron spectroscopy (XPS). The surface morphologies were examined using scanning electron microscopy (SEM). Dental glass–ceramics were tested in constant immersion, 3-day cycling, and 1-day cycling with pH 10, pH 2, and pH 7 for 3, 15, and 30 days. The 1-d cycling group demonstrated the highest levels of weight loss compared with 3-d cycling and constant immersion. For the ion release, Si^4+^ and Ca^2+^ had the highest rates of release in 1-d cycling, whereas the Al^3+^ release rate with constant pH 2 was highest. The alteration/passivation layer that was formed on the surface of disks possibly prevented further dissolution of pH 10 corroded disks. XPS analysis demonstrated different surface compositions of corroded disks in pH 10 and pH 2. Si^4+^, K^+^, Na^+^, Al^3+^, and Ca^2+^ were detected on the surface of corroded pH 10 disks, whereas a Si^4+^ and P^5+^-rich surface formed on corroded pH 2 disks. SEM results demonstrated rougher surfaces for corroded disks in cycling conditions and pH 2 constant immersion. In conclusion, increased pH changes significantly promote the corrosion of dental glass–ceramic materials.

## 1. Introduction

Glass–ceramic materials are used to veneer prosthetic restorations for oral rehabilitation. Ceramic restorative materials are esthetic with good translucency and light transmission that enable a good match with tooth shades. Ceramics are also wear-resistant and biocompatible [1,2,3]. However, these restorations have a higher failure rate than metal restorations in numerous clinical studies. Systematic reviews showed survival rates were 88% to 97% for ceramic prostheses, compared with 94% to 97% for metal prostheses for 3 to 5 years [4,5,6,7,8]. The survival rates decreased to 22% to 47% [9,10] for ceramic prostheses and 74% to 85% for metal prostheses for 13 to 15 years [11,12,13,14].

Ceramics were thought to be inert [15] but are now known to be susceptible to corrosion [16,17]. In the oral environment, dietary pH fluctuates from acidic to alkaline depending on personal dietary preferences. Research studies reported different pH levels in food, drinks, and fruits [18,19,20,21], which could initiate the potential dissolution of teeth or the corrosion of ceramic materials. Despite the buffering capacity of saliva and cleansing ability of the tongue, the oral cavity is a harsh environment for restorative dental materials. A clinical study showed a roughening of ceramic surfaces caused by corrosion, wear, or both [22]. The roughening of ceramic surfaces is detrimental to the surface integrity of the opposing enamel antagonist [23] and adversely affects the fracture strength of the restoration [24,25]. International Standards Organization (ISO) standard 6872 tests for dental ceramic chemical durability [26] and constantly immerses ceramic in 4% acetic acid at 80 °C for 16 h. However, several studies demonstrated the effect of different pH solutions on the surface degradation and corrosion of ceramic [20,21,27,28]. Firouz et al. showed orange juice (pH 3.5) and cola (pH 2.4) increased the surface roughness of ceramic [20]. Esquivel-Upshaw et al. demonstrated that ceramics corroded in low and high pH solutions through different mechanisms [21]. Additionally, another study demonstrated that cyclic pH solutions between acidic and alkaline, akin to pH changes in the mouth, resulted in an increased surface degradation compared with just constant immersion [27]. With the variation in dietary pH, the current ISO 6872 [26] test for chemical durability may be underestimating the corrosive effect of the oral environment on ceramic restorations. The aim of this study was to test the hypothesis that the increased frequency of pH cycling would result in increased ceramic corrosion as a function of weight loss and ion release.

## 2. Experimental

### 2.1. Sample Preparation

Fluorapatite glass ceramic veneer disks (E.max Zirpress, Ivoclar Vivadent AG, Schaan, Liechtenstein, 12.6 × 1.7 ± 0.3 mm) were used. The disks were polished on both sides through 340, 400, and 600 grits (Carbimet, Buehler, Lake Bluff, IL, USA), cleaned using ethanol under ultrasonic, and rinsed by deionized distilled (DI) water. The compositions of disks are listed in Table 1.

### 2.2. Experimental Design

The disks were divided into 3 groups: (1) constant immersion, (2) 3-day (3-d) cycling, and (3) 1-day (1-d) cycling. Three disks were used for each condition. All disks were dried at 100–105 °C in the oven for 24 h and cooled down in the desiccator before the weight measurement. The balance with an accuracy of 0.1 mg was used for the weight measurement (AS60/220.R2 Analytical balance, RADWAG).

The disks were immersed in pH 10 (potassium carbonate–potassium borate–potassium hydroxide buffer, SB116-500, Fisher Chemical, Pittsburgh, PA, USA), pH 2 (potassium chloride–hydrochloric acid, SB96-500, Fisher Chemical, Pittsburgh, PA, USA), and pH 7 (potassium phosphate monobasic–sodium hydroxide, SB108-500, Fisher Chemical, Pittsburgh, PA, USA) buffer solutions. The cycling sequence was pH 10, pH 2, then pH 7 for both cycling groups. For the 3-d cycling, the disks were immersed in pH 10 for the first 3 days, pH 2 for the next 3 days, pH 7 for the next 3 days, and the entire sequence was repeated up to 30 days. For 1-d cycling, the immersion period was only 1 day for each pH following the same sequence of pH 10, 2, 7. Disks were analyzed at 3, 15, and 30 days. Cycling disks were also analyzed at 27 days, as this time period represents an equal number of days per pH for 3-d cycling, i.e., 3 cycles each for pH 2, 7, and 10.

All the disks were placed in polyethylene centrifuge tubes (Thermo Scientific Nalgene Oak Ridge High-Speed Centrifuge Tubes, Thermo Fisher Scientific, Waltham, MA, USA), which were placed in a shaking water bath (Water bath shaking TSBS40, Techne, Vernon Hills, IL, USA) with 80 °C and 50 oscillations per minute.

### 2.3. Characterizations

The weight measurement was performed prior to and after the experiment. The levels of Si^4+^, Al^3+^, Ca^2+^, and Zn^2+^ ions released in solutions were analyzed using inductively coupled plasma atomic emission spectrometer (ICP, 3200RL, PerkinElmer, Waltham, MA, USA). K^+^ and Na^+^ ions were not analyzed because of their presence in the buffer solutions. Analysis of variance (ANOVA) was used to estimate the effects of treatment, time, and the interaction between treatment and time on cumulative ion release. For all models, the base-2 log of cumulative ion release was taken as the outcome in order to meet linear modeling assumptions. All analyses were performed using the R statistical software package (V.4.0.0, R Foundation for Statistical Computing, Vienna, Austria). The surface composition of non-corroded disks and corroded disks was analyzed using an X-ray photoelectron spectroscopy instrument (ULVAS-PHI XPS, ULVAC-PHI, Kanagawa, Japan) with Al monochromatized Kα radiation from a 50 W X-ray source. The surface morphology of constant immersion, 3-d cycling, and 1-d cycling disks was analyzed using scanning electron microscopy (SEM, Tescan Mira3, Kohoutovice, Czech Republic). Platinum was sputter coated on the disks prior to SEM analysis.

## 3. Results

### 3.1. Weight Loss

**Figure 1:**
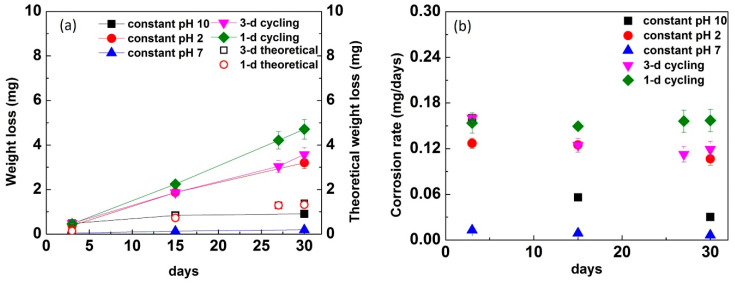
The (**a**) weight loss and (**b**) corrosion rate of constant immersion in pH 10, pH 2, and pH 7 at 3-d cycling and 1-d cycling for 3 days, 15 days, and 30 days. The theoretical weight loss for 3-d cycling and 1-d cycling is also shown.

The weight loss of the disks from constant immersion and pH cycling is shown in Figure 1a, and the corrosion rate in mg/days was calculated in Figure 1b. For constant immersion, the weight loss was highest in pH 2, followed by pH 10 and pH 7 across all time points (Figure 1a). In addition, the weight loss appeared to be constant despite an increase in immersion time among all the constant conditions. For the cycling groups, the results displayed that 1-d cycling had greater weight loss compared with the 3-d cycling (*p* = 0.87). The theoretical weight loss for 3-d and 1-d cycling was calculated by using the weight loss of constant immersion for 30 days for each pH divided by 30 and multiplied by the number of days cycled. Then, the number of cycles per pH were added to total 30 days immersion. The theoretical weight loss of cycling groups on average demonstrated lower weight loss than the experimental results. Comparing cycling groups and constant groups, both cycling groups showed significantly higher weight loss than constant groups (*p* = 0.0001 for both 1-d cycling and 3-d cycling).

In Figure 1b, the corrosion rate in pH 10 constant immersion was highest at 3 days and decreased with an increase in immersion time. The corrosion rate for constant immersion in pH 10 at 30 days was almost the lowest among the groups with the exception of constant immersion in pH 7. The corrosion rate for pH 2 was lower than pH 10 at 3 days and was similar with 15 days, and the rate decreased slightly at 30 days. For constant pH 7 immersion, the corrosion rate was the lowest among all the test conditions. For 3-d cycling, the corrosion rate decreased slightly as the time increased. The corrosion rate became similar to constant pH 2 after 15 days, but the rate was still higher than constant pH 10. For 1-d cycling, the corrosion rate was similar across all time points and maintained the highest corrosion rate compared with all of the test conditions across all time points.

### 3.2. ICP Analysis

**Figure 2:**
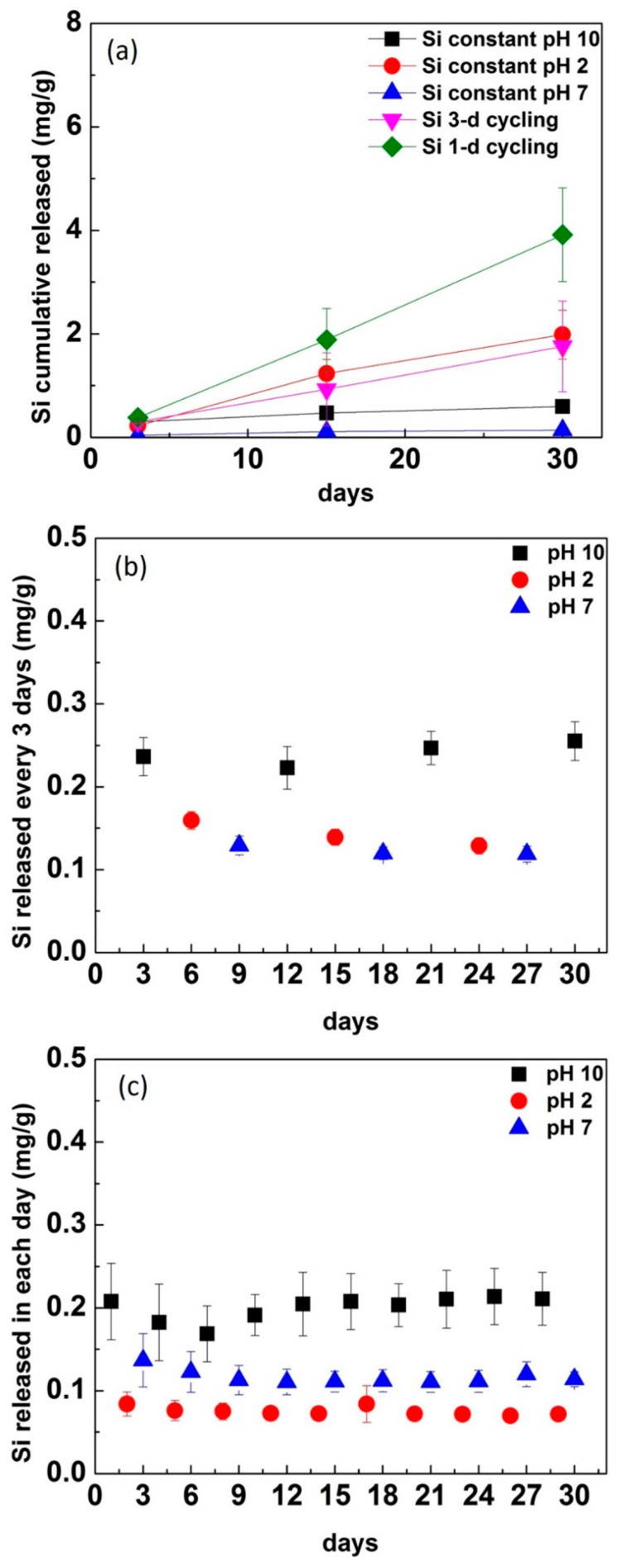
The cumulative Si^4+^ ion released in each condition (**a**) and the Si^4+^ released in each pH and time individually in (**b**) 3-d cycling and (**c**) 1-d cycling.

For constant immersion, the cumulative release of Si^4+^ ions was highest in pH 2, then pH 10, and followed by pH 7 across all time points (Figure 2a). Comparing cycling with constant immersion, 1-d cycling showed more Si^4+^ ion release at 15 days, whereas 3-d cycling was almost the same as pH 2 constant immersion for the same time point. At 30 days, 1-d cycling had almost twice Si^4+^ release than constant immersion, whereas 3-d cycling was slightly less than pH 2 constant immersion. Si^4+^ had a significantly higher rate of release at 1-d cycling than 3-day cycling (*p* < 0.0001), and constant immersion in pH 10 (*p* < 0.0001) and pH 7 (*p* < 0.0001). While there was no significant difference in the rate of increase between 1-d cycling and constant pH 2, 1-d cycling had a significantly higher mean release across all time points (*p* = 0.012).

The released Si^4+^ at each pH and time point for both 1-d and 3-d cycling groups is also shown individually (Figure 2b,c). The released Si^4+^ was highest in pH 10 for 3-d cycling and 1-d cycling. The released Si^4+^ in pH 2 from 3-d cycling was lower than in pH 10 but slightly higher than in pH 7. However, the released Si^4+^ in pH 2 from 1-d cycling was lowest for each individual release compared with pH 10 and pH 7 (Figure 2c).

**Figure 3:**
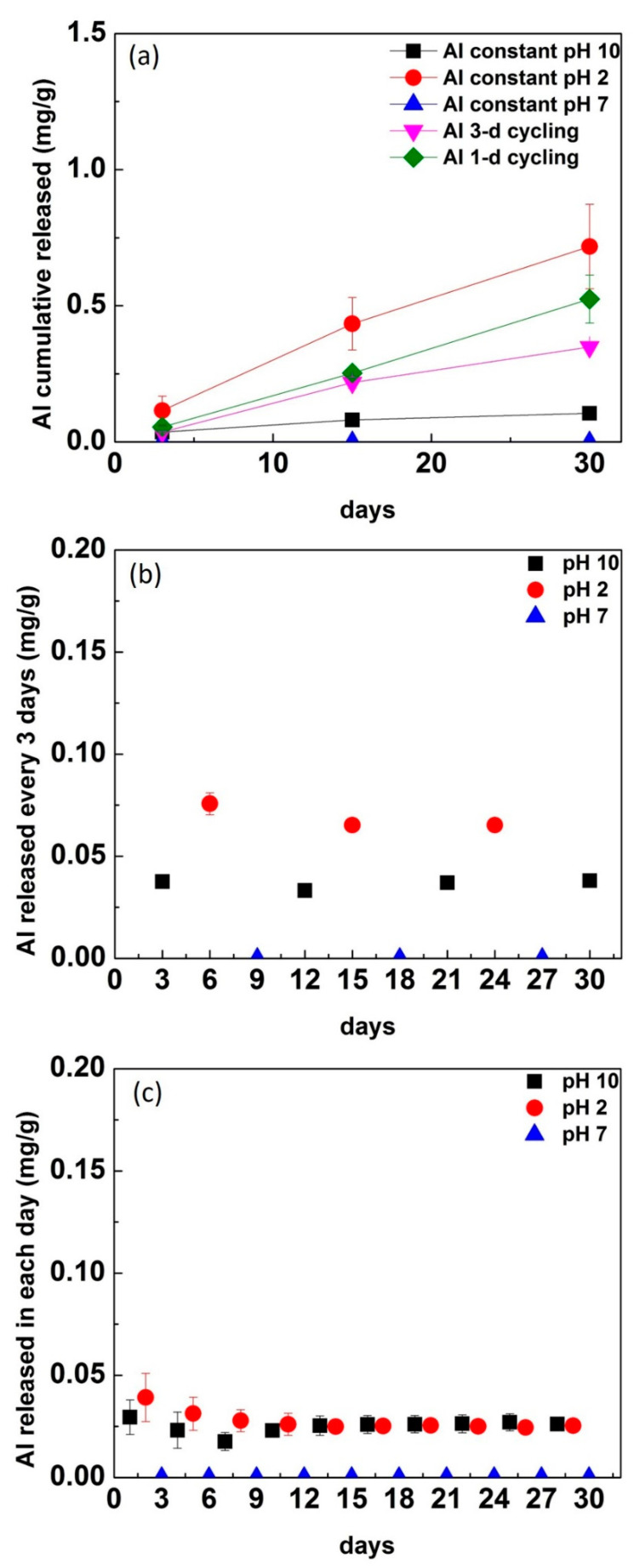
The cumulative Al^3+^ ion released in each condition (**a**) and the Al^3+^ released in each pH and time individually in (**b**) 3-d cycling and (**c**) 1-d cycling.

Al^3+^ showed a higher cumulative release in pH 2 than pH 10, and it was not detectable in pH 7 at constant 15-day immersion. Cycling cumulative release results were similar between 3-d cycling and 1-d cycling at 15 days. Comparing cycling and constant immersion, the cumulative Al^3+^ ion released from cycling groups was less than in constant pH 2 immersion but higher than constant pH 10 (Figure 3a). After 30 days, Al^3+^ had a higher cumulative release in constant pH 2 immersion, with minimal release in pH 10 constant immersion, and it was not detectable in pH 7. The 1-d cycling release was higher than 3-d cycling, but both were less than constant pH 2 immersion. The Al^3+^ release rate in 1-d cycling (*p* < 0.0001), 3-d cycling (*p* = 0.001), and constant pH 2 (*p* = 0.044) was significantly higher than constant pH 10. While there was no significant difference in the rate of increase between 1-d cycling, 3-d cycling, and constant pH 2 (*p* > 0.05), pH 2 had a significantly higher mean release than 1-d cycling (*p* = 0.003), 3-day cycling (*p* = 0.0004), and constant pH 10 (*p* = 0.0002).

The released Al^3+^ values in the cycling groups at each time period are shown individually (Figure 3b,c). The released Al^3+^ was higher in pH 2 than in pH 10 in both cycling groups and was not detected in pH 7.

**Figure 4:**
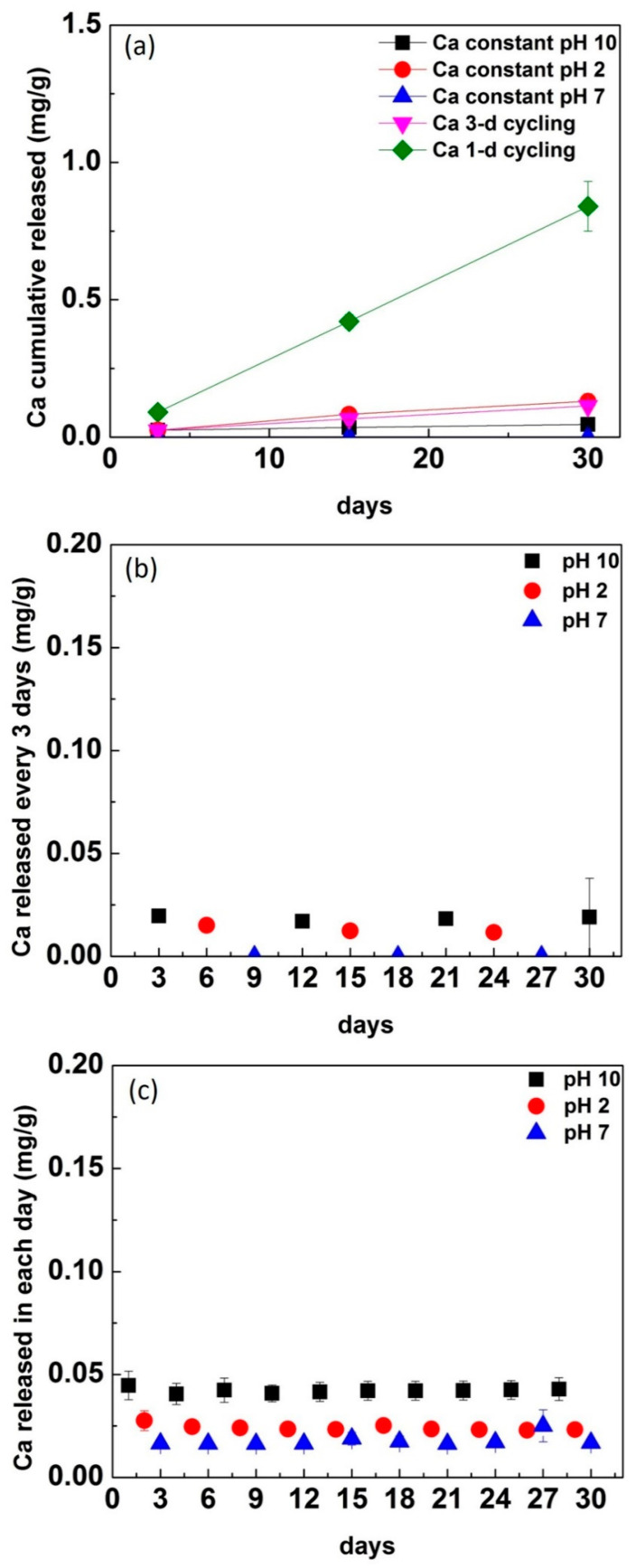
The cumulative Ca^2+^ ion released in each condition (**a**) and the Ca^2+^ released in each pH and time individually in (**b**) 3-d cycling and (**c**) 1-d cycling.

Ca^2+^ ions showed little to no detected release among constantly immersed conditions. However, Ca^2+^ was released cumulatively in 1-d cycling for 15 days and continued to increase release up to 30 days (Figure 4a). Ca^2+^ had a significantly higher rate of release in 1-d cycling than 3-d cycling (*p* < 0.0001), and constant immersion in pH 10 (*p* < 0.0001) and pH 2 (*p* = 0.0004).

Ca^2+^ release in 1-d cycling at each time point is almost twice as high as release in 3-d cycling; Ca^2+^ release is highest in pH 10, followed by pH 2, and was almost undetectable in pH 7 (Figure 4b,c). Zn^2+^ was almost undetectable in constant and cycling conditions. Therefore, the result of Zn^2+^ was not discussed and presented.

### 3.3. XPS Analysis

The reference and corroded disks were analyzed using XPS. The surface compositions in atomic percentage are listed in Table 2. In constant immersion in pH 10, Si^4+^, Al^3+^, Na^+^, K^+^ and Ca^2+^ were detected on the surface for the 3-day and 30-day time points. In pH 2, there was mainly Si^4+^ on the surface for 3 days. After 30 days, Si^4+^, P^5+^, and Zr^4+^ were detected, and there was no Al^3+^, Na^+^, K^+^, and Ca^2+^ on the surface.

### 3.4. SEM Analysis

**Figure 5:**
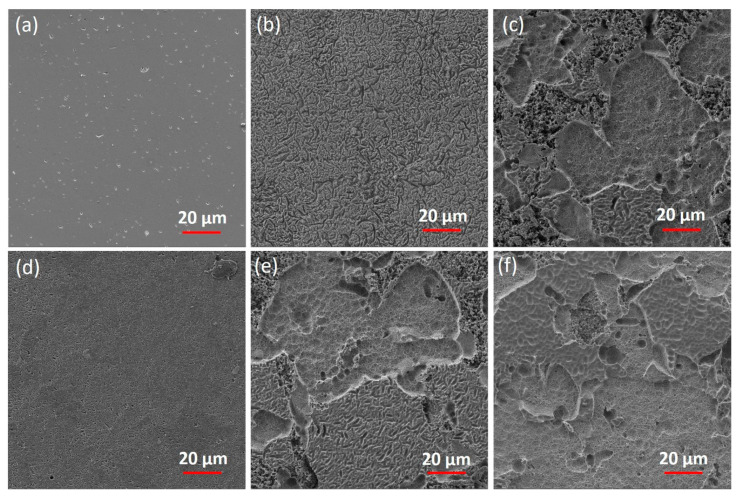
The SEM images of (**a**) reference (baseline) and corroded disks for 30 days of immersion in constant (**b**) pH 10, (**c**) pH 2, (**d**) pH 7, and (**e**) 3-d cycling and (**f**) 1-d cycling.

The surface morphologies of the reference and corroded disks were examined using SEM (Figure 5). The corroded disks showed different surface morphologies from the reference disk. After corrosion, a generalized pitting surface was observed in pH 10 (Figure 5b) and pH 7 (Figure 5d) solutions, where the pitting was larger in pH 10 than in pH 7. On the other hand, the isolated areas of the corroded surface were observed in pH 2 (Figure 5c) and 3-d and 1-d cycling conditions (Figure 5e,f, respectively).

## 4. Discussion

This study aimed to test if the increased frequency of pH changes would accelerate ceramic corrosion. Since different food and drinks with a variety of pH levels are constantly introduced in the mouth and are constantly being buffered by saliva, increased pH changes were performed to simulate the frequency of these changes.

Glasses and ceramics have been reported to form a thin layer on the surface during the corrosion process [30,31,32]. This film is called a passivating film or alteration layer, and has limited further degradation of ceramics and played an important role in the material’s durability. The formation of an alteration or passivation layer on a material susceptible to corrosion has acted as a barrier to minimize corrosion and played a major role in corrosion resistance. A previous study described three general characteristic stages that occur in corrosion [32]: (1) the *initial stage* where the corrosion rate is at the maximum at a given pH and temperature; (2) the *residual stage* where the alteration layer is formed and the corrosion rate is slow and nearly constant; and (3) the *resumption stage* where the corrosion rate increases again. The 1-d cycling demonstrated the highest corrosion rate across all time points (Figure 1b). The 1-d cycling experiment is considered to be the initial stage for all time points, where the solution remained fresh, dilute, and flowing. The glass–ceramic was exposed to the new solution every day, and the corrosion process was initiated with hydroxyl ions (OH^−^) and protons (H^+^) with the maximum corrosion rate. On the other hand, the corrosion rates in the constant pH 10 and pH 2 immersion conditions decreased with time. During the corrosion process, the ions were released from the surface into solution, and the concentration of released ions increased, while the corrosion rate decreased. The alteration layer formed on the surface can interfere with the hydroxyl ions or protons being diffused from solution to the surface or the ions released from the surface into solution. As the time increased, the interference of the alteration layer on dissolution became more significant. The corrosion rate of 3-d cycling was lower than that of 1-d cycling at 15 days and 30 days. This might indicate that the alteration layer was beginning to form during the 3-day period.

Studies have demonstrated differences in the glass–ceramic corrosion reactions while immersed in different pH environments [27,28]: (1) in basic solutions, the glass network former, SiO_4_^2−^, undergoes total dissolution with the cleavage of SiO_4_^2−^ bonds by hydroxyl ions in basic solution; (2) in acidic solutions, the network modifiers (alkali metal and alkali earth metal) are ion-exchanged with hydrogen ions or protons in solution, and released into solution; (3) in neutral solutions, there is a combination of total dissolution and ion exchange reactions.

From the ICP results, the released level of Si^4+^ in pH 10 was higher than pH 2 in cycling conditions (Figure 2b,c). This could be explained by the dissolution of SiO_4_^2−^ in solution because of the nucleophilic attack through hydroxyl ions [27,28]. In contrast, for constant immersion conditions, the alteration layer formed with time prevented ions from being further released from the surface into solution. The Si^4+^ ions were released much slower and in smaller quantities in constant pH 10 than constant pH 2 (Figure 2a). The Si^4+^ showed the lowest release in constant pH 7, where the total dissolution (hydroxyl ions) and ion exchange process (hydrogens or protons) occur simultaneously. In the 1-d cycling, the Si released level in pH 10 for 1 day was almost the same as the Si^4+^ released in pH 10 at 3 days from the 3-d cycling. This might indicate the beginning formation of the alteration during the 3-day period. The 1-day cycling did not demonstrate any effect of the alteration layer. The glass–ceramic showed higher Si^4+^ ion release in pH 10 followed by pH 7 and pH 2.

The cumulative release of Al^3+^ in pH 2 was higher than in pH 10 (Figure 3). A possible explanation is that the alumina compound is known for having a small ionization constant in pH 10 solution, where a lower release of Al^3+^ ions was observed [33]. In pH 2, the corrosion of glass–ceramic was dominated by hydrogen ions, where the activity of hydrogen ions was higher and favored an ionic exchange reaction [27,28]. The Al^3+^ released in pH 2 was through an ionic exchange reaction with hydrogen ions in solution. Ca^2+^, a network modifier, was released in higher amounts in constant pH 2 than pH 10. In contrast, Ca^2+^ was released in higher amounts in pH 10 cycling than pH 2 cycling conditions (Figure 4). A possible explanation is that the alteration layer was formed on the interface in constant pH 10 condition and prevented ion release into solution (Figure 4a). In cycling conditions, the formation of an alteration layer is disrupted with the changes in pH leading to a higher release of Ca^2+^. With the cycling frequency increased from 3-d to 1-d cycling, the released Ca^2+^ at each day was higher in 1-d cycling compared with 3-d cycling (Figure 4b,c). In addition, some network modifiers were released in solution during dissolution, whereas some network modifiers remained in the surface layer. They were further released in pH 2 (new environment) through ion exchange. This might explain why the released level of Ca^2+^ was slightly less in pH 2 than in pH 10 in cycling groups.

The surface of corroded samples was analyzed using XPS. Si^4+^, Al^3+^, Na^+^, K^+^, and Ca^2+^ were detected on the surface of corroded disks constantly immersed in pH 10 (Table 2). These results are in agreement with the ICP results, which demonstrated less release of Si^4+^, Al^3+^, and Ca^2+^ in pH 10 constant immersion condition with the possible formation of an alteration layer [31]. There was a slight increase of Na^+^ and K^+^ on the pH 10 corroded surface. These ions could be used for charge compensation, while Al^3+^ was slightly increased and Ca^2+^ slightly decreased. The presence of Ca^2+^ either from solution or glass could enhance the passivating properties of the alteration layer [30]. In this study, the released Ca^2+^ ions in solution might interact with the silica-rich surface and become integrated into the alteration layer, where the Ca^2+^ in the solution from 3-d cycling at each time point was lower than that from 1-d cycling (Figure 4b,c). Ca^2+^ was detected on the pH 10 corroded surface (Table 2). In pH 2, Al^3+^, Na^+^, K^+^, and Ca^2+^ ions were not detected from the 3-day corroded disks, and a Si-rich hydrated surface seems to have formed (Table 2). This could be explained by the ion exchange that occurs in an acidic environment, where Al^3+^, Na^+^, K^+^, and Ca^2+^ are released from the surface into solution. These results are in agreement with ICP results, which showed more ions released in constant immersion in pH 2 than in pH 10. With an increase in time, more ions were released into solution. P, Ti, and Zr remained on the surface and detected. The Si and P-rich hydrated surface could interfere with the degradation process in the long term and become the rate-limiting step in corrosion in an acidic environment [27,28].

SEM results demonstrated the surface morphologies of corroded disks to be rougher in cycling conditions and pH 2 compared with corroded disks in pH 10, pH 7, and reference disks (Figure 5). These results are in agreement with total weight loss results (Figure 1), where cycling conditions and pH 2 had higher weight loss compared with other groups. The corroded pH 10 disks showed less surface roughness, which might indicate formation of the alteration layer on the surface, which limited the degradation of ceramic.

Results from this experiment demonstrated that increasing the frequency of pH changes through a pH cycling mechanism results in increased ion release and weight loss from glass–ceramic materials compared with a constant immersion protocol. This could indicate that current in vitro tests for chemical durability could be underestimating the corrosion in the oral environment.

## 5. Conclusions

This study demonstrated that the increased frequency of pH changes significantly enhanced the corrosion of glass–ceramic. The alteration layer significantly affected the corrosion processes by hindering the release of surface ions into solution. The methodology of cycling pH with consistent pH changes disrupted the formation of an alteration layer and resulted in more weight loss and ion release during these cycling conditions. The glass–ceramic exhibited less chemical durability during cycling immersion. This testing methodology reflected a more realistic simulation of the oral environment with vacillating pH caused by the consumption of foods with different pH levels. The conventional standard testing of constant immersion for dental chemical durability might be underestimating the corrosion, which occurs intraorally.

## Figures and Tables

**Table 1:** The compositions of the E.max Zirpress glass–ceramic disks used in this study [29].

Composition	SiO_2_	Al_2_O_3_	Na_2_O	K_2_O	CaO	ZnO	ZrO_2_	P_2_O_5_	F	Other Oxides	Pigments
wt %	57.0–62.0	12.0–16.0	7.0–10.0	6.0–8.0	2.0–4.0		1.5–2.5	1.0–2.0	0.5–1.0	0–6.0	0.2–0.9
atomic%	58.6–51.6	14.5–15.7	13.9–16.1	7.8–8.5	2.2–3.5		0.7–1.0	0.4–0.7	1.6–2.6		

**Table 2:** The surface composition (atomic %) of reference, corroded disks in pH 10 and pH 2 for 3 days and 30 days of constant immersion.

Atomic Ratio	Si	Al	Na	K	Ca	Mg	P	Zn	Zr	N	F	Ti
ref	53.7	11.5	7.6	5.9	3.5	3.3	1.3	1.3	0.6	9.9	1.4	
pH 10, 3 d	60.0	13.9	13.9	9.3	2.9							
pH 10, 30 d	59.8	15.8	9.5	12.0	2.9							
pH 2, 3 d	94.8								5.2			
pH 2, 30 d	56.0						19.4		17.7			6.9
